# Programme-level determinants of participation in colorectal cancer screening in Italy: a national cross-sectional study

**DOI:** 10.1186/s12889-026-28525-4

**Published:** 2026-07-17

**Authors:** Riccardo Marmo, Ludovica Venezia, Francesca Galeazzi, Renato Cannizzaro, Marco Soncini

**Affiliations:** 1 Gastroenterologia, PO Polla, ASL Salerno, Salerno, Italy; 2 SC Gastroenterologia, Azienda Ospedaliero-Universitaria Maggiore della Carità, Novara, Italy; 3 Gastroenterologia, Azienda Ospedaliera di Padova, Padova, Italy; 4 Oncological Gastroenterology Unit, Centro di Riferimento Oncologico Aviano (CRO) IRCCS, via Franco Gallini 2, Aviano (PN), 33081 Italy; 5 Department of Medical, Surgical and Health Sciences, University of Trieste, Trieste, Italy; 6 Dipartimento Area Medica, ASST Lecco, Lecco, Italy

**Keywords:** Colorectal cancer screening, Participation rate, Organisational determinants, Health services research, Faecal immunochemical test, Population-based screening, Screening programmes

## Abstract

**Background:**

Participation in organised colorectal cancer (CRC) screening is influenced by multiple organisational factors; however, existing evidence has mainly focused on isolated interventions, with limited understanding of how these components jointly influence participation within real-world screening settings. This study aimed to identify the organisational determinants of participation in population-based CRC screening in Italy and to quantify their relative contribution within a real-world, decentralised healthcare system, hypothesising that participation is determined by the effect of multiple organisational domains rather than by isolated interventions.

**Methods:**

We conducted a national cross-sectional survey of Italian CRC screening programmes using a validated web-based questionnaire. Fifty-eight programmes from 17 regions were included. The outcome was the adjusted participation rate in 2022. Associations between organisational characteristics and participation were assessed using multiple fractional logistic regression models.

**Results:**

The participating services covered a target population of 5.9 million individuals. Participation varied substantially across settings (range 6.5%-67.1%). In multivariable analysis, higher participation was independently associated with Northern geographic area (OR 2.03; 95% CI 1.73–2.38; *p* < 0.001), programme duration > = 16 years (OR 1.87; 95% CI 1.28–2.73; *p* < 0.01), the presence of a unified regional management system (OR 1.45; 95% CI 1.08–1.94; *p* = 0.01), coordinated communication of positive faecal immunochemical test (FIT) results (OR 1.44; 95% CI 1.13–1.84; *p* = 0.003), communication by general practitioners (OR 1.35; 95% CI 1.06–1.72; *p* = 0.014), and structured invitation procedures including educational materials and reminders. These findings suggest that participation is shaped by the effect of multiple organisational components rather than by isolated interventions.

**Conclusions:**

This study provides a system-level evaluation of organisational determinants of CRC screening participation and quantifies their relative contribution within real-world programmes. The proposed framework may support screening services in identifying priority organisational domains for intervention and in designing integrated strategies tailored to their operational context.

**Supplementary Information:**

The online version contains supplementary material available at https://doi.org/10.1186/s12889-026-28525-4.

## Background

 Colorectal cancer (CRC) remains a major cause of cancer-related morbidity and mortality worldwide, and its burden is expected to increase in the coming decades [1]. Population-based screening is an effective strategy to reduce CRC incidence and mortality through early detection and removal of precancerous lesions [2]. However, participation in screening remains suboptimal and highly variable across settings and healthcare systems [3, 4].

A substantial body of literature has investigated determinants of screening uptake, identifying both individual and organisational factors. Most studies, however, have focused on single interventions, such as reminders, educational materials, or improved access, evaluated in isolation [5–7]. While these approaches provide important evidence, they do not fully capture the complexity of real-world screening delivery, where multiple organisational components interact within specific healthcare settings.

In particular, there is limited evidence on how different organisational domains jointly influence participation and on the relative contribution of these components within integrated screening systems. This gap is especially relevant in decentralised healthcare systems, where programme performance may vary substantially depending on governance models, organisational structure, and resource allocation.

Understanding participation in organised screening therefore requires a system-level perspective that considers the combined effect of organisational characteristics rather than isolated interventions. Such an approach may provide more actionable insights for service optimisation and for the design of context-specific strategies aimed at improving uptake.

We hypothesised that participation in CRC screening programmes is jointly determined by multiple, organisational domains – rather than by single interventions evaluated independently – and that their relative contribution can be quantified within a real-world, decentralised healthcare system.

The aim of this study was to identify organisational determinants of participation in population-based colorectal cancer screening in Italy and to quantify their relative impact within a real-world, decentralised healthcare system.

## Methods

### Study design

A cross-sectional observational study was conducted through the administration of a structured questionnaire to 174 Italian gastroenterology centres involved in the organisation and delivery of colorectal cancer screening. The survey aimed to collect information on organisational factors that may influence participation of the target population in screening. Each participating centre was represented by a single responding unit; the terms centre, unit, and programme are therefore used interchangeably throughout the manuscript to refer to the same entity, namely the responding service and the organisational screening programme operating within the area it serves.

The Italian National Health Service (SSN) guarantees, in principle, universal and free access to essential healthcare services, including organised cancer screening, under a single national law defining the essential levels of care (Livelli Essenziali di Assistenza, LEA). Colorectal cancer screening is part of the National Prevention Plan and is implemented at the regional level: each of the 20 Italian regions enacts its own regional legislation within this national framework and is responsible for organising, managing, and monitoring its own screening programme, which may include a dedicated regional coordination structure overseeing local screening activities delivered by individual health authorities and gastroenterology centres. Within each region, broader sub-regional territorial areas may further adapt regional legislation to local needs. This multi-level, decentralised organisational model can result in substantial variability in invitation procedures, kit delivery and return modalities, and management of FIT results across regions and centres, even within a single national health system.

### Questionnaire development and validation

The study was conducted as a cross-sectional survey using the CAWI (Computer-Assisted Web Interviewing) methodology for data collection. This approach is widely recognised in the literature for healthcare surveys, as it allows large samples to be reached within a short time and at relatively low cost [8, 9].

In a preliminary phase, a subgroup of five centres was involved in development of the questionnaire. Through virtual meetings and email consultations, proposals for items and suggestions were collected and used to draft the first version of the questionnaire. This collaborative process ensured content validity and alignment with the operational needs of the participating centres [10].

A run-in phase was subsequently implemented, during which the preliminary version of the questionnaire was tested in a small sample to assess clarity, internal consistency, and technical functionality of the web platform. Feedback obtained during this phase was used to refine the instrument.

After the run-in phase, the questionnaire underwent external validation, including expert review to ensure content validity [10], reliability analysis through calculation of internal consistency using Cronbach’s alpha for the relevant scales [11], and a comprehension test consisting of pre-administration to a small sample to ensure clarity and interpretability of the questions [12]. These procedures resulted in minor revisions, ensuring that the questionnaire represented a valid and reliable tool for data collection.

Each centre provided information on the regional organisational setting and, more specifically, on the organisation of local screening activities, including invitation procedures, kit distribution and return modalities, and management of faecal immunochemical test (FIT) results. The reference year for evaluation of participation was 2022.

### Definitions

Screening invitation was defined as the formal and systematic activity through which the screening programme contacts eligible individuals by written, electronic, or telephone communication, providing information on the procedure, free access, location, and purpose of the test.

Adjusted participation rate was defined as the proportion of invited individuals who accepted the invitation and actually underwent the screening test using the following formula: number of tests performed / number of valid invitations sent x 100, excluding undelivered invitations and subjects with a recent FIT or colonoscopy.

The target age range was defined as the age interval used for systematic invitation to screening for both sexes.

A programme update was defined as any modification or organisational change implemented within the previous five years in the screening programme of the territory served by the participating centre.

Unified regional coordination was defined as the presence of a single dedicated centre, or physical structure, responsible for coordinating screening activities across the region. A unified regional management system was defined as the presence of a single, region-wide operational system (e.g., a shared information platform or centralised administrative procedures) for managing screening activities, as opposed to independent management by individual sub-regional territorial units.

Communication of positive kit result was defined as the modality through which the responsible structure (endoscopy unit, local health authority centre, regional coordination centre, or general practitioner) actively contacted the participant to inform them of a positive FIT result.

### Questionnaire administration

After validation, the questionnaire was distributed by email to all centres involved in colorectal cancer screening in Italy between 15 January 2024 and 15 March 2024. Each centre received a unique link to access the online questionnaire, allowing response tracking and monitoring of response rates.

The platform allowed completion from different devices, including computer, tablet, and smartphone, and included a function for saving partial responses, enabling participants to complete the questionnaire in multiple sessions.

During the data collection period, reminder emails were sent at regular intervals to encourage participation. Data collection form did not include any centre-identifying information; responses allowed data to be aggregated by geographic area only; the anonymised dataset was transferred to the statistical analysis team, ensuring that no participating programme could be identified and that the confidentiality of the collected information was preserved.

### Use of artificial intelligence tools

A generative artificial intelligence tool was used solely to assist with language editing and preparation of graphical material. The authors critically reviewed and validated all AI-assisted output and take full responsibility for the final content of the manuscript.

### Statistical analysis

All statistical analyses were performed at programme level (*n* = 58), corresponding to the subset of the 174 contacted gastroenterology centres for which complete questionnaire data were obtained (see Results); population figures are provided for descriptive purposes only. Descriptive statistics were used to summarise sample characteristics. Continuous variables were expressed as means +/- standard deviations or medians with interquartile ranges and were compared using Student’s t test or the Mann-Whitney U test, as appropriate. Categorical variables were compared using the chi-square test or Fisher’s exact test, according to expected cell frequencies.

Population figures were reported to describe the size and regional coverage of the participating centres and were not used as weights in the statistical analyses.

The adjusted participation rate was analysed as a continuous proportion (0–1) and treated as the dependent variable. Multiple fractional logistic regression models were used to estimate the independent association between participation and each variable, adjusting simultaneously for the set of independent variables, grouped into the following domains: organisational setting, invitation modality, kit delivery, kit return, and management of FIT results.

The internal consistency of the questionnaire was assessed by calculating Cronbach’s alpha coefficient. Missing data were handled by listwise deletion. All statistical analyses were performed using Stata version 19.1 (StataCorp, College Station, TX, USA).

## Results

The national survey conducted by the Associazione Italiana Gastroenterologi Ospedalieri (AIGO) involved 174 gastroenterology centres, of which 60 (34.5%) responded and 58 (33.3%) provided complete data, representing 17 of the 20 Italian regions. The annual target population served by the participating centres was 5,913,562 residents, of whom 4,687,190 (79.3%) received an invitation letter; the remaining 20.7% either did not receive an invitation, because it was not sent by the programme, or could not be reached due to undelivered invitations, most frequently caused by incorrect or outdated address information and, less frequently, by changes in the recipient’s residence or domicile; among these, 1,529,829 adhered to the invitation (32.6%), and 78,312 tested positive (5.1%) (Table 1).

**Table 1:** Descriptive characteristics of programmes participating in the survey

	Italy (all programmes)	Programmes participating in the study			
		Italy	North	Centre	South-Islands
Programmes (n)	106	58	25	11	22
Annual target population	8,561,549	5,913,562	2,230,460	1,760,005	1,923,097
Invited population	7,085,308	4,687,190	2,647,898	816,007	1,223,285
Number of participants	2,316,700	1,529,829	1,069,779	222,958	237,092
Adjusted participation*	32.7	32.6	46.4	28.7	20.9

* Percentage of invited subjects who performed the test, excluding undelivered invitations and subjects with a recent FIT or colonoscopy

*Abbreviations*: *FIT* Faecal immunochemical test

In 30 centres (51.7%), the target age range was 50–74 years; in 19 (32.7%), 50–69 years; and in 9 (15.5%), 50–70 years. The test used for faecal occult blood detection was the faecal immunochemical test (FIT) in 56 centres (96.6%). A cut-off value of at least 80 µg/dL was adopted in all except one.

**Fig. 1:**
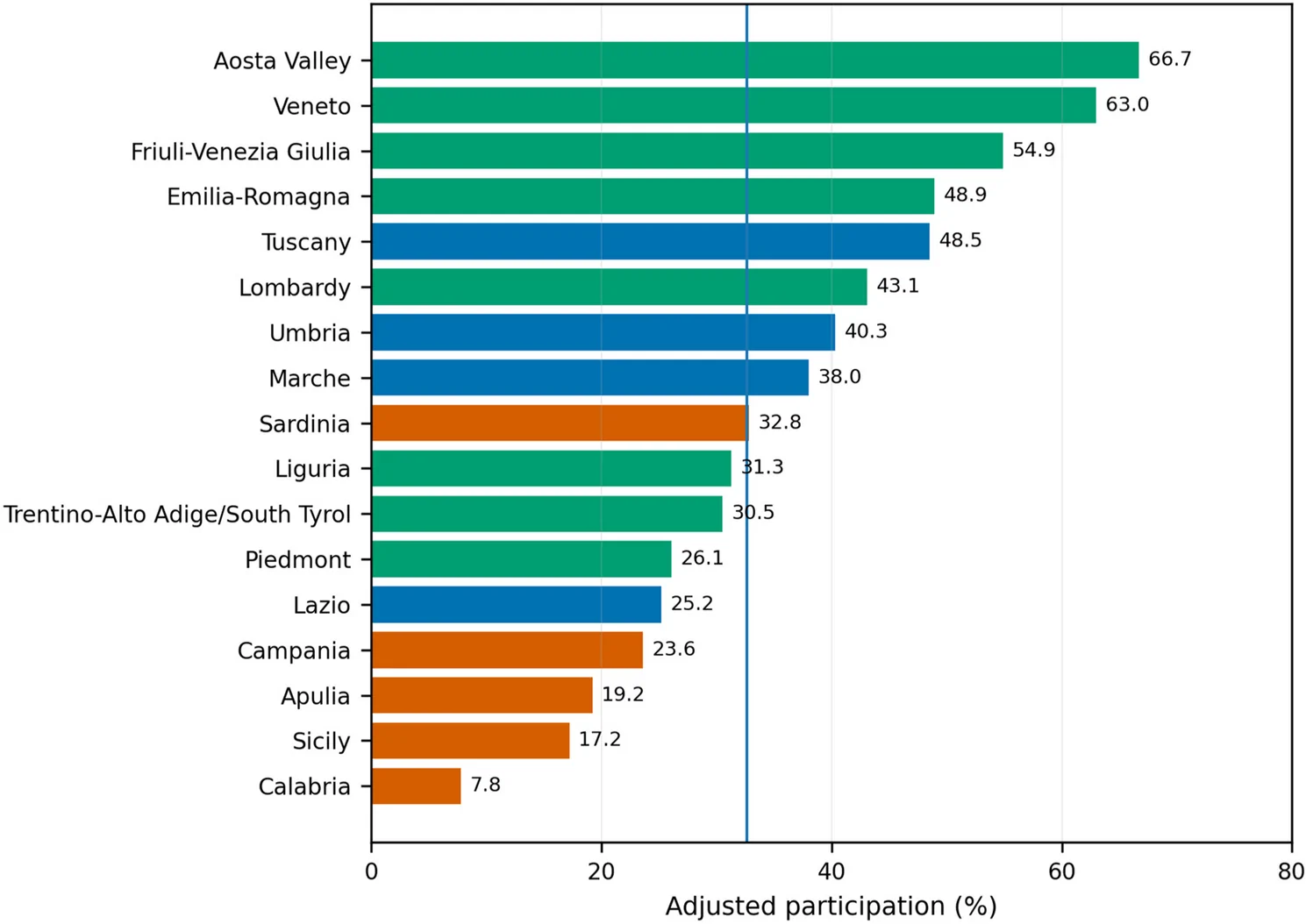
Adjusted participation in colorectal cancer screening across Italian regions in 2022. Bar colours indicate geographic macro-area: green, Northern regions; blue, Central regions; orange, Southern and Island regions

Screening adjusted participation rates showed substantial heterogeneity. In 2022, participation in colorectal cancer screening in Italy displayed marked inter-regional variability, with a clear geographic pattern. The lowest adjusted participation rate (6.5%) was recorded in the area served by a centre in Sicily, whereas the highest was observed in Veneto (67.1%). The overall mean adjusted participation rate ranged from 7.8% to 66.7% across regions, with a national mean adjusted participation rate of 32.6% (*p* < 0.001). Screening services operating in Southern and Island regions, including Calabria, Sicily, Campania, and Puglia, showed the lowest levels, generally below 25%, whereas those in Northern regions, including Emilia-Romagna, Veneto, Friuli-Venezia Giulia, and Valle d’Aosta, and in Tuscany reported significantly higher values, exceeding 48% (*p* < 0.001) (Fig. 1).

FIT positivity was also heterogeneous (Supplementary Fig. 1) and showed an inverse pattern compared with participation. FIT positivity ranged from 2.6% to 14.5% (Italy: 5.1%; *p* < 0.001). Screening services in Calabria, Sicily, Campania, and Puglia reported the highest positivity rates (> 7%), whereas those in Marche, Trentino, Veneto, and Liguria showed lower values (< 4%) (*p* < 0.001).

### Organisational determinants and participation rate

#### Geographic macro-area

Geographic area was a significant source of heterogeneity in both univariate and multivariate analyses. Mean participation was 46.4% (95% CI 38.9–53.9) in Northern programmes, 28.7% (95% CI 22.3–35.2) in Central Italy, and 20.9% (95% CI 16.2–25.6) in Southern and Island regions (*p* < 0.001) (Table 2).

**Table 2:** Organisational characteristics of colorectal cancer screening programmes and participation in response to the invitation

Characteristic	Programmes (*n*)	Participation mean [95% CI]	*p* value*
Macro-area			
South-Islands	22	20.9 [16.2, 25.6]	0.0001
Centre	11	28.7 [22.3, 35.2]	
North	25	46.4 [38.9, 53.9]	
Unified regional coordination			
No	9	21.2 [14.4, 28.0]	0.0006
Yes	49	36.3 [31.9, 40.8]	
Unified regional management system			
No	17	26.9 [20.8, 33.0]	0.0121
Yes	41	36.9 [31.9, 42.0]	
Target age			
50–69	19	30.1 [24.7, 35.4]	0.4164
50–70	9	35.6 [18.7, 52.5]	
50–74	30	36.0 [30.1, 41.9]	
Management software updates during the last 5 years			
No	26	27.6 [22.7, 32.6]	0.0031
Yes	32	39.2 [33.3, 45.0]	
Years of programme activity			
1–8	14	25.9 [19.3, 32.4]	0.0001
8–12	16	28.3 [23.0, 33.6]	
12–16	14	32.9 [23.9, 42.0]	
16–30	14	49.7 [41.6, 57.8]	
Presence of a regional coordination centre dedicated to colorectal screening in the area			
No	19	30.2 [22.4, 38.0]	0.2159
Yes	39	35.82 [30.9, 40.7]	

* Mean differences were assessed using one-way analysis of variance (ANOVA)

Geographic area remained significantly associated with participation after adjustment in the multivariate model.

#### Organisational setting

The organisational setting influenced participation rates. In services where duration was at least 16 years, participation was 49.7% (95% CI 41.6–57.8), significantly higher than in those with shorter duration (*p* < 0.001). The presence of a unified management system was another independent factor positively associated with participation (Table 2).

#### Invitation modality

Sending the invitation letter together with an explanatory brochure and subsequently one or two reminders was positively associated with participation (Tables 3 and 4). Reminders should be sent within 90 days of the initial invitation. Invitations delivered by email or telephone appeared to be the least effective modalities (Table 3).

**Table 3:** Invitation modalities for colorectal cancer screening and participation

Characteristic	Programmes (*n*)	Participation mean [95% CI]	*p* value*
Invitation modality (multiple options possible)			
Email	2	24.8	0.4510
Telephone	2	24.2 [143.1, 191.6]	
Paper-based	46	35.6 [30.9, 40.3]	
Paper-based + mail, telephone, SMS	8	29.4 [16.5, 42.2]	
Invitation letter includes an explanatory brochure			
No	18	24.3 [18.8, 29.8]	0.0003
Yes	40	38.4 [33.4, 43.3]	
Reminder system			
No	27	27.6 [23.1, 32.2]	0.0023
Yes	31	39.5 [33.4, 45.6]	
Number of reminders			
0	27	27.6 [23.1, 32.2]	0.0323
1	20	39.3 [31.1, 47.5]	
2	9	40.4 [28.9, 51.9]	
3	2	38.3 [-204.7, 281.3]	
Reminder within days			
1–30	5	34.0 [13.8, 54.2]	0.1234
30–90	16	44.9 [35.3, 54.5]	
>90	5	27.2 [8.1, 46.4]	

* Mean differences were assessed using one-way analysis of variance (ANOVA)

*Abbreviations*: *SMS* Short message service

**Table 4:** Multivariable analysis of factors associated with the degree of participation in colorectal cancer screening

Variable / Level	Odds ratio	95% CI	*p* value
Macro-area			
South	Ref		
Centre	1.33	1.05–1.69	0.018
North	2.03	1.73–2.38	0.000
Unified regional coordination	1.18	0.90–1.55	0.221
Unified regional management system	1.45	1.08–1.94	0.014
The screening programme has undergone updates	1.09	0.84–1.41	0.528
Years of programme presence			
1–8	Ref		
8–12	1.30	0.96–1.77	0.092
12–16	1.23	0.82–1.85	0.323
16–30	1.87	1.28–2.73	0.001
Presence of brochure in the invitation	1.19	0.89–1.58	0.244
Reminder system			
None	0.92	0.70–1.20	0.534
1	Ref		
2	1.13	0.90–1.41	0.299
3	0.62	0.41–0.92	0.019
Kit delivery at			
Pharmacy	Ref		
Health district	0.71	0.43–1.18	0.188
Home delivery	1.09	0.67–1.75	0.733
Pharmacy HD	0.74	0.53–1.02	0.068
Pharmacy GP	1.34	0.94–1.92	0.105
Pharmacy home delivery	0.73	0.51–1.04	0.078
HD GP	0.88	0.49–1.56	0.654
Pharmacy HD GP	0.70	0.48–1.00	0.052
Pharmacy HD home delivery	0.82	0.62–1.10	0.188
Communication of positive kit result			
Endoscopy	Ref		
Local health authority centre	1.44	1.13–1.84	0.003
Regional centre	0.93	0.59–1.44	0.732
GP	1.35	1.06–1.72	0.014

*Abbreviations*: *CI* Confidence interval, *GP* General practitioner, *HD* Health district, *Ref* Reference category. Kit delivery channels (pharmacy, health district, general practitioner, and home delivery) were not mutually exclusive; categories denote the specific combination of channels available within each programme

#### FIT kit distribution

Distribution of the FIT kit through pharmacies, home delivery, general practitioners, or mixed models combining these approaches was associated with higher participation (Supplementary Table 1, Table 4).

#### FIT kit return

Return of the FIT kit through pharmacies was the most frequently used modality. Return via postal services, general practitioners, or mixed models showed numerically higher participation rates; however, differences between modalities were not statistically significant (p = 0.56) (Supplementary Table 2).

### Communication of results

No significant differences were observed among the different modalities used to communicate negative FIT results (p = 0.40). The adjusted participation rate was significantly associated with the modality used to communicate positive results (p < 0.007). Higher adjusted participation was observed in programmes where positive results were communicated by the local coordination centre (OR 1.44; 95% CI 1.13-1.84; p = 0.003) or by the general practitioner (OR 1.35; 95% CI 1.06-1.72; p = 0.014) (Table 4; Supplementary Table 3).

## Discussion

Importantly, our findings extend previous evidence by moving beyond the evaluation of single interventions and adopting a system-level perspective that captures the complexity of real-world screening systems.

Italy operates within a unified national regulatory framework. However, the 20 Regions have substantial organisational and managerial autonomy in healthcare. As a consequence, although operating within a common legal framework, each Region can independently define the organisational structure of screening services. This autonomy is reflected in the variability of regional performance observed in colorectal cancer screening, as shown by our results. Participation rates were significantly heterogeneous, and FIT positivity also varied across regions, displaying a pattern complementary to that of participation.

High FIT positivity was inversely associated with participation and tended to decrease as programme duration increased. Our findings are consistent with previous evidence showing a decline in FIT positivity across successive screening rounds (rate ratio 0.56; 95% CI 0.53-0.60) [13].

Regional governance represents a major determinant of participation. The results of this survey indicate that organisational and structural factors strongly influence the effectiveness of colorectal cancer screening services. Among organisational variables, the presence of a regional screening system active for at least 16 years and the availability of a unified regional management system were the most significant factors. Individuals who participate in previous screening rounds have a significantly higher probability of re-participating compared with those who have never participated, and regular invitation-reinvitation systems increase cumulative participation [14–16]. Programme duration can therefore be considered a proxy for the number of completed screening rounds and for progressive optimisation of organisational processes.

This proxy effect likely reflects several converging elements: accumulated organisational experience, progressive refinement of procedures, improvement in the quality of informational materials over time, increased local awareness and reputation of the programme within its territory, and the cumulative inclusion of repeat participants from previous invitation rounds. Several of these individual elements, such as the presence of an explanatory brochure, were independently associated with participation in univariate analysis (Table 2); however, in the multivariable model, programme duration emerged as the most relevant independent variable, likely capturing and subsuming the effect of these related organisational components.

Invitation to screening accompanied by an explanatory brochure represents an important determinant of participation and should be followed by a reminder system, with no more than two reminders sent within 90 days of the initial invitation. Several studies have confirmed the effectiveness of reminder systems and patient navigation interventions in improving adherence to colorectal cancer screening [17–19]. Although previous studies have demonstrated the effectiveness of individual interventions, their impact is highly context-dependent and may vary according to the organisational setting in which they are implemented.

Distribution of the FIT kit through pharmacies or general practitioners was associated with the highest participation levels. Direct mailing of the FIT kit to the patient's home may represent an additional strategy to increase participation, particularly among vulnerable populations. In low-income healthcare settings in the United States, active mailing of FIT kits accompanied by educational material significantly improved participation rates compared with usual care, resulting in higher FIT completion than reminder letters alone [20].

The modalities of FIT kit return analysed in our study were not significantly associated with differences in participation rates. Telephone reminders represent a distinct organisational lever, separate from kit return modality; other studies have shown that telephone reminders at two weeks can improve FIT return rates by approximately 10% [20, 21].

Communication of test results by the local screening centre or by the general practitioner occurs downstream of participation itself, but it may contribute to a positive perception of the screening service, which takes responsibility for patient management and organises further diagnostic procedures. International recommendations support multicomponent models tailored to individual preferences and local context [2, 22, 23]. By simultaneously evaluating multiple and independent organisational domains, this study allows estimation of their relative contribution, providing insights that cannot be derived from studies focusing on isolated components.

To our knowledge, this is one of the few studies evaluating organisational determinants of CRC screening participation within a comprehensive programme-level framework rather than as isolated interventions.

### Strengths

This study provides a system-level evaluation of organisational determinants within a single national health system, allowing comparison across regions operating under the same regulatory framework but with different governance models.

The analysis included programmes covering approximately 69% of the Italian target population and captures the multiple organisational components, reflecting real-world programme complexity rather than isolated interventions. These findings offer evidence relevant to decentralised healthcare systems, where organisational variability may substantially influence screening performance.

### Limitations

Several limitations should be considered. First, data were self-reported and may be subject to reporting bias. Second, the response rate was moderate (34%), raising the possibility of selection bias if higher-performing programmes were more likely to participate; however, the participating programmes covered approximately 69% of the national target population, supporting representativeness. Third, the analysis was conducted at programme level and did not account for individual-level determinants of participation. Due to the cross-sectional design, causal relationships cannot be established, and the findings should be interpreted as associations rather than causal effects. Finally, the questionnaire explored organisational characteristics at a macro-system level and did not capture specific operational interventions, such as mass communication campaigns or appointment-based invitations.

### Future perspectives

Future research should integrate individual-level data with population-based analyses to assess applicability of this organisational model in other national and international settings. Experimental studies evaluating targeted organisational interventions in the most critical domains could further improve understanding of factors influencing participation in screening programmes.

## Conclusions

Participation in colorectal cancer screening is determined by the overall organisational configuration of screening services rather than by single interventions. Strategies including unified governance, structured invitation systems, and coordinated communication pathways are associated with improved participation. By quantifying the relative impact of multiple organisational components within real-world settings, this study provides a practical framework to support programme optimisation and the design of new screening strategies. These findings are particularly relevant for decentralised healthcare systems, where organisational variability strongly influences screening performance. Importantly, these findings suggest that the effectiveness of individual interventions cannot be fully interpreted without considering the organisational context in which they are implemented. By providing a system-level assessment of organisational determinants, this study offers a practical framework to support optimisation of existing screening services and the design of evidence-based screening strategies.

## Supplementary Information

Supplementary Material 1.

## Data Availability

The datasets generated and/or analysed during the current study are available from the corresponding author on reasonable request.

## Abbreviations

AIGO: Italian Association of Hospital Gastroenterologists and Endoscopists
CAWI: Computer-Assisted Web Interviewing
CRC: Colorectal cancer
FIT: Faecal immunochemical test
SSN: Italian National Health Service
