# Supplementary material for: Programme-level determinants of participation in colorectal cancer screening in Italy: a national cross-sectional study
Source: BMC Public Health. 2026 Jul 17;26:2945. doi: 10.1186/s12889-026-28525-4 (PMC13660311; doi:10.1186/s12889-026-28525-4)
Supplement: Supplementary file 1 [file 12889_2026_28525_MOESM1_ESM.docx]

**Supplementary Figure 1. FIT positivity across Italian regions in 2022.**

FIT positivity was calculated as the proportion of individuals with a positive faecal immunochemical test among those who performed the test. Bar colours indicate geographic macro-area: green, Northern regions; blue, Central regions; orange, Southern and Island regions.

**Supplementary Table 1. Kit delivery modalities and participation in colorectal cancer screening**

| **Variable / Factor** | **Programmes (n)** | **Participation (mean) [95% CI]** | **p value*** |
| --- | --- | --- | --- |
| Kit delivery |  |  |  |
| Pharmacy | 26 | 41.0 [34.4, 47.6] | 0.089 |
| Pharmacy + Home delivery | 4 | 38.1 [22.8, 53.5] |  |
| Home delivery | 4 | 34.5 [28.6, 40.4] |  |
| Pharmacy + GP | 1 | 31.6 |  |
| Pharmacy + Health district | 11 | 27.9 [17.4, 38.3] |  |
| Health district + GP | 2 | 24.8 |  |
| Pharmacy + Health district + GP | 3 | 24.6 [3.7, 45.5] |  |
| Health district | 4 | 23.4 [-6.2, 52.9] |  |
| Pharmacy + Health district + Home delivery | 3 | 20.1 [16.5, 23.7] |  |

Abbreviations: GP, general practitioner; HD, health district.
* Mean differences were assessed using one-way analysis of variance (ANOVA).

**Additional file 1 - Supplementary Table 2. Kit return modalities and participation in colorectal cancer screening**

| **Variable / Factor** | **Programmes (n)** | **Participation (mean) [95% CI]** | **p value*** |
| --- | --- | --- | --- |
| Kit return |  |  |  |
| Pharmacy | 24 | 39.6 [32.7, 46.5] | 0.564 |
| Pharmacy + Postal service | 2 | 43.5 [20.4, 66.7] |  |
| GP | 1 | 38.0 |  |
| Postal service | 2 | 34.4 [-4.5, 73.2] |  |
| Health district + Postal service | 1 | 31.3 |  |
| Pharmacy + Health district | 11 | 29.4 [18.5, 40.4] |  |
| Health district | 13 | 28.9 [19.4, 38.4] |  |
| Pharmacy + Health district + GP | 2 | 26.1 [-75.4, 127.6] |  |
| Health district + GP | 1 | 24.8 |  |
| Pharmacy + Health district + Postal service | 1 | 20.1 |  |

Abbreviations: GP, general practitioner; HD, health district.
* Mean differences were assessed using one-way analysis of variance (ANOVA).

**Additional file 1 - Supplementary Table 3. Communication of FIT results and participation in colorectal cancer screening**

| **Variable / Factor** | **Programmes (n)** | **Participation (mean) [95% CI]** | **p value*** |
| --- | --- | --- | --- |
| Communication of test result |  |  | 0.405 |
| Regional screening centre | 17 | 38.6 [31.4, 45.9] |  |
| Health district | 6 | 33.6 [17.4, 49.8] |  |
| Local screening centre | 34 | 32.2 [26.5, 37.9] |  |
| General Practitioner | 1 | 18.1 |  |
|  |  |  |  |
| In case of positive result |  |  |  |
| Endoscopy service | 33 | 28.1 [23.1, 33.1] | 0.007 |
| Local Health Authority centre | 16 | 42.9 [35.7, 50.1] |  |
| Regional centre | 7 | 39.7 [25.2, 54.2] |  |
| General Practitioner | 2 | 39.9 [15.3, 64.6] |  |

Abbreviations: FIT, faecal immunochemical test; GP, general practitioner; HD, health district; ASL/AUSL, local health authority.
* Mean differences were assessed using one-way analysis of variance (ANOVA).
